# Heart rate, mortality, and the relation with clinical and subclinical cardiovascular diseases: results from the Gutenberg Health Study

**DOI:** 10.1007/s00392-019-01466-2

**Published:** 2019-04-05

**Authors:** Thomas Münzel, Omar Hahad, Tommaso Gori, Sebastian Hollmann, Natalie Arnold, Jürgen H. Prochaska, Andreas Schulz, Manfred Beutel, Norbert Pfeiffer, Irene Schmidtmann, Karl J. Lackner, John F. Keaney, Philipp S. Wild

**Affiliations:** 1 Center for Cardiology – Cardiology I, University Medical Center of the Johannes Gutenberg-University Mainz, Langenbeckstraße 1, 55131 Mainz, Germany; 2 Center for Thrombosis and Hemostasis, University Medical Center of the Johannes Gutenberg-University Mainz, Mainz, Germany; 3 Partner site RhineMain, German Center for Cardiovascular Research (DZHK), Mainz, Germany; 4 Preventive Cardiology and Preventive Medicine, Center for Cardiology, University Medical Center of the Johannes Gutenberg-University Mainz, Mainz, Germany; 5 Department of Psychosomatic Medicine and Psychotherapy, University Medical Center of the Johannes Gutenberg-University Mainz, Mainz, Germany; 6 Department of Ophthalmology, University Medical Center of the Johannes Gutenberg-University Mainz, Mainz, Germany; 7 Institute of Medical Biostatistics, Epidemiology and Informatics, University Medical Center of the Johannes Gutenberg-University Mainz, Mainz, Germany; 8 Institute of Clinical Chemistry and Laboratory Medicine, University Medical Center of the Johannes Gutenberg-University Mainz, Mainz, Germany; 9 Division of Cardiovascular Medicine, University of Massachusetts Medical School, Worcester, MA USA

**Keywords:** Heart rate, Mortality, Vascular (endothelial) function, Neurohumoral biomarkers, Population-based

## Abstract

**Background:**

Higher, but also lower resting heart rate (HR), has been associated with increased cardiovascular events and mortality. Little is known about the interplay between HR, cardiovascular risk factors, concomitant diseases, vascular (endothelial) function, neurohormonal biomarkers, and all-cause mortality in the general population. Thus, we aimed to investigate these relationships in a population-based cohort.

**Methods:**

15,010 individuals (aged 35–74 at enrolment in 2007–2012) from the Gutenberg Health Study were analyzed. Multivariable regression modeling was used to assess the relation between the variables and conditional density plots were generated for cardiovascular risk factors, diseases, and mortality to show their dependence on HR.

**Results:**

There were 714 deaths in the total sample at 7.67 ± 1.68 years of follow-up. The prevalence of diabetes mellitus, arterial hypertension, coronary and peripheral artery disease, chronic heart failure, and previous myocardial infarction exhibited a J-shaped association with HR. Mortality showed a similar relation with a nadir of 64 beats per minute (bpm) in the total sample. Each 10 bpm HR reduction in HR < 64 subjects was independently associated with increased mortality (Hazard Ratio 1.36; 95% confidence interval 1.06–1.75). This increased risk was also present in HR > 64 subjects (Hazard Ratio 1.29; 95% confidence interval 1.19–1.41 per 10 bpm increase in HR). Results found for vascular and neurohormonal biomarkers exhibited a differential picture in subjects with a HR below and above the nadir.

**Discussion:**

These results indicate that in addition to a higher HR, a lower HR is associated with increased mortality.

**Electronic supplementary material:**

The online version of this article (10.1007/s00392-019-01466-2) contains supplementary material, which is available to authorized users.

## Background

For many years, increased heart rate (HR) has been demonstrated to be associated with increased cardiovascular (CV) mortality in patients with coronary artery disease (CAD) [1] and with chronic heart failure (CHF) [2]. Data from the Framingham Heart Study also suggest that mortality rates increase progressively in relation to HR [3]. This relation persisted after excluding individuals with pre-existing CV disease [3], suggesting HR was not simply a marker of previous cardiac damage. Conversely, pharmacological HR reduction with agents such as the I_f_-channel inhibitor, ivabradine, has been shown to reduce angina symptoms in patients with CAD [4] and to improve both congestive symptoms as well as prognosis in patients with CHF [5].

Based on this literature, it has been proposed that an elevated HR may be considered an independent CV risk factor [6]. This notion must be tempered by evidence that traditional risk factors such as smoking, obesity, arterial hypertension, and diabetes mellitus or prevalent CV disease such as CHF with reduced or preserved ejection fraction have also been linked to elevated HR [7, 8]. Nevertheless, HR reduction strategies have gained traction when associated with favorable outcomes as demonstrated by the inclusion of ivabradine in the European Society of Cardiology Treatment Guidelines for CAD and CHF [9, 10]. In contrast to the literature outlined above, recent studies in CAD patients have called into question the benefits of pharmacologic HR reduction with compounds such as ivabradine. Indeed, the SIGNI_f_Y study, that treated CAD patients with ivabradine (7.5 mg bid) reported an increase in CV events (HR of 60.7 beats per minute (bpm) in the ivabradine group versus 70.6 bpm in the placebo group) [11].

These conflicting data with ivabradine question whether the specific extent of HR reduction is important in determining benefit versus harm. Thus, we sought to address this question in the Gutenberg Health Study (GHS), a prospective cohort study of 15,010 individuals, subjected to intense characterization of both clinical and emerging functional CV risk factors.

## Methods

### Study design and sample

The GHS is a population-based, prospective single-center cohort study in Midwestern Germany with a total sample size of 15,010 individuals. The study design has been published elsewhere in detail [12]. Briefly, individuals between the ages of 35 and 74 years were drawn randomly from local governmental registries with a sampling procedure that was stratified for sex, residential area (urban versus rural), and decades of age. Participant recruitment began in April 2007 and was completed in April 2012. The baseline investigation involved a highly standardized 5-h clinical examination at the study center performed by specifically trained and certified medical technical assistants according to a uniform written protocol. Informed consent was obtained from all individual participants included in the study and all procedures performed in the GHS were approved by the ethics committee of the Statutory Physician Board of the State of Rhineland-Palatinate and the local data safety commissioners. The study design is in accordance with the revised Helsinki protocol and principles outlined in recommendations for Good Clinical and Epidemiological Practice.

### Heart rate assessment

Resting HR was measured by an oscillometric technique (using OMRON HEM model 705IT, OMRON Medizintechnik, Germany) under standardized conditions. Specifically, measurements were performed in a quiet environment in the sitting position with uncrossed legs and open eyes after 5, 8, and 11 min of rest while remaining quiet. The mean of the 2nd and 3rd measurements was used as the resting HR. In cases of atrial fibrillation (AF), results were compared with those determined automatically via a 12-lead resting ECG (GE Healthcare, CardioSoft v6). ECG-based diagnosis of AF was made by two cardiologists. We observed a close correlation in the total sample (*r* = 0.79) and in cases of AF (*r* = 0.76) between the two methods (Online Resource 1).

### Definitions of cardiovascular risk factors and laboratory methods

Comprehensive information on CV risk factors was gathered by means of standardized interviews, anthropometric measures, and laboratory assessments. Concomitant disease and medication history were derived from a standardized interview and from medical records. Venous blood was obtained after an overnight fast (at least 8 h) before vascular function measurement. Samples were processed for plasma and stored in aliquots at − 80 °C immediately after blood draw. We used routine laboratory methods for blood glucose and lipid measurements. Plasma CT-proAVP, MR-proADM, MR-proANP, endothelin-1, and serum NT-proBNP and troponin I were determined using commercially available assays according to the manufacturer’s instructions as reported previously [13]. Reproducibility was good, with all of the coefficients of variation (intraassay and interassay) < 5%. The definitions of CV risk factors and biomarker measurements are described elsewhere in detail [13–16].

### Determination of vascular (endothelial) function by flow-mediated dilation and peripheral arterial tonometry

FMD of the brachial artery was measured after a 5-min upper arm occlusion as percentage increase of brachial artery diameter in resting condition according to guidelines as described [13, 17]. Brachial artery two-dimensional high-resolution ultrasound images were acquired with a Philips HD11XE CV ultrasound machine (Best, The Netherlands) using a L12-5 (38 mm) linear array broadband probe. Artery diameters were measured offline using the commercially available Brachial Analyzer software tool, version 5.0 (Medical Imaging Applications LLC, Iowa City, IA, USA).

Regarding peripheral arterial tonometry, pneumatic pulse amplitude was measured with the Endo-PAT2000 device (Itamar Medical, Caesarea, Israel) and digital volume pulse was registered electronically with a PulseTrace 2000 device (Micro Medical Limited, Rochester, United Kingdom) analyzing waveform automatically as described [13, 15, 17]. Therefore, resistance artery endothelial function was estimated by reactive hyperemia index (RHI), small artery vascular tone by reflection index (RI), and systemic large artery stiffness as stiffness index (SI) as well as augmentation index (AI).

Measurements of FMD and peripheral arterial tonometry were performed simultaneously and further details and quality control data about the good reproducibility of the methods in the GHS have been described previously [13, 15, 17].

### Data management and statistical analysis

All data of the present investigation underwent quality control by a central data management unit. Data were reviewed for completeness by predefined algorithms and plausibility criteria. Mortality updates were performed by quarterly queries to the registry offices and the mortality registry Rhineland-Palatinate. For death reviews official death certificates were acquired.

Study sample characteristics are presented by 1-standard deviation (SD) (11 bpm) below and above the nadir of HR (as subsequent analyses showed the lowest mortality at a resting HR of 64 bpm) as absolute and relative frequency for categorical variables and as mean value and SD or median with 25th and 75th percentiles for continuous variables. Conditional density plots were generated for CV risk factors, concomitant CV diseases, and mortality to show their relative frequencies in dependence on HR. To assess the associations between HR, CV risk factors, CV diseases, and markers of subclinical disease, linear regression models with multivariable adjustment were used. All models are presented with the adjusted variables. Medications were screened as three-digit categories of the Anatomical Therapeutic Chemical Classification (ATC) [18] in a linear regression model for HR with adjustment for age and sex. Medication classes associated with a *P* value < 0.01 were taken in the regression models as relevant confounders. For mortality analyses, Cox proportional hazards regression models were used. All effect estimates are given with 95% confidence interval (95% CI) and *P* value. Because of the explorative nature of the study, no Bonferroni correction of *P* values was conducted. *P* values should be treated as a continuous measure of statistical strength of an association and they are, therefore, reported exactly. All tests were two sided and *P* values < 0.05 were considered significant. Statistical data analyses were performed using the software program R, version 3.3.1 (http://www.R-project.org).

## Results

### Relation between heart rate and sample characteristics

As evident in Table 1, with increasing HR there was a trend to a higher proportion of women. Most traditional CV risk factors showed two maxima below and above the nadir of HR, except obesity and smoking that exhibited a continual increase with higher HR. This was also true for CV diseases, except CAD and myocardial infarction (MI), which were continuously less frequent with higher HR.

**Table 1:** Sample characteristics according to groups of heart rate

Groups of heart rate at rest			
Heart rate [bpm]	< 53	53–75	> 75
*N*	720	10,327	3,878
Sex (Women)	32.1% (231)	49.1% (5068)	53.0% (2054)
Age (years)	56.9 ± 11.1	54.9 ± 11.1	54.7 ± 11.1
*Cardiovascular risk factors*			
Diabetes mellitus	8.6% (62)	8.0% (822)	12.9% (499)
Arterial hypertension	53.1% (382)	46.5% (4799)	57.6% (2234)
Smoking	17.8% (128)	19.3% (1993)	20.1% (779)
Obesity	22.6% (163)	22.8% (2350)	32.1% (1243)
Dyslipidemia	48.7% (350)	43.2% (4449)	46.4% (1791)
Family history of MI/stroke	23.6% (170)	22.0% (2272)	22.2% (860)
*Concomitant disease*			
CHF	9.2% (66)	7.0% (720)	9.3% (359)
CAD	9.6% (68)	4.5% (461)	2.8% (107)
MI	6.1% (44)	3.0% (310)	2.1% (83)
Stroke	2.1% (15)	1.8% (187)	1.9% (74)
AF	18.5% (133)	16.5% (1699)	16.6% (643)
PAD	4.4% (31)	3.1% (315)	4.0% (153)
COPD	3.3% (24)	4.7% (481)	6.1% (238)
CKD	0.8% (6)	1.0% (102)	1.1% (43)
*Surrogate marker for cardiovascular disease*			
Heart rate [bpm]	49.2 ± 3.0	65.1 ± 5.9	82.9 ± 7.2
BMI (kg/m^2^)	27.2 ± 4.3	27.1 ± 4.7	28.1 ± 5.6
SBP (mmHg)	131.3 ± 17.4	130.4 ± 17.2	134.8 ± 17.7
DBP (mmHg)	77.7 ± 8.7	81.5 ± 9.1	86.2 ± 9.6
MAP (mmHg)	95.6 ± 10.2	97.8 ± 10.8	102.4 ± 11.4
HbA1c (%)	5.57 ± 0.56	5.54 ± 0.65	5.67 ± 0.82
LDL (mg/dl)	132.7 ± 33.5	138.8 ± 35.2	140.6 ± 36.4
HDL (mg/dl)	55.9 ± 14.9	57.7 ± 15.6	56.7 ± 15.8
Triglycerides (mg/dl)	122.6 ± 65.8	120.2 ± 71.4	133.7 ± 98.1
FMD (%)	7.09 ± 4.44	8.18 ± 5.19	8.30 ± 5.54
Baseline brachial artery diameter	4.57 ± 0.82	4.32 ± 0.85	4.28 ± 0.86
SI (m/s)	7.30 ± 2.36	7.53 ± 2.26	7.63 ± 2.03
RI	74.72 ± 15.89	67.10 ± 15.42	58.80 ± 15.64
AI (%)	31.37 ± 25.13	19.30 ± 20.70	9.53 ± 15.69
RHI	0.62 ± 0.42	0.65 ± 0.42	0.65 ± 0.41
Baseline pulse amplitude	620.2 ± 471.0	568.3 ± 444.9	546.6 ± 407.0
Troponin I > 0.02* (ng/mL)	6.9% (18)	3.2% (106)	4.5% (57)
Endothelin 1* (pmol/l)	59.4 (50.9/68.3)	58.9 (50.2/67.7)	59.3 (51.0/69.2)
CT-proAVP* (pmol/l)	2.75 (1.75/4.25)	2.73 (1.79/4.31)	2.96 (1.79/4.84)
MR-proADM* (nmol/L)	0.47 (0.40/0.56)	0.46 (0.39/0.53)	0.47 (0.40/0.57)
NT-proBNP* (pg/mL)	86.47 (39.88/192.43)	62.44 (29.48/125.57)	55.95 (24.06/109.96)
MR-proANP* (pmol/l)	89.2 (65.8/121.0)	67.6 (50.7/91.0)	57.0 (43.1/78.6)

Plus–minus values are means ± standard deviation and two values in parentheses are medians with 25th and 75th percentiles

*CHF* chronic heart failure, *CAD* coronary artery disease, *MI* myocardial infarction, *AF* atrial fibrillation, *PAD* peripheral artery disease, *COPD* chronic obstructive pulmonary disease, *CKD* chronic kidney disease, *BMI* body mass index, *SBP* systolic blood pressure, *DBP* diastolic blood pressure, *MAP* mean arterial pressure, *LDL* low-density lipoprotein, *HDL* high-density lipoprotein, *FMD* flow-mediated dilation, *SI* stiffness index, *RI* reflection index, *AI* augmentation index, *RHI* reactive hyperemia index

*Biomarkers were available in *N* = 5,000

To visualize the relation between HR and prevalence of CV risk factors (Online Resource 2) and diseases (Online Resource 3), conditional density plots were generated for the prevalence of each variable over the HR range. We found diabetes mellitus and arterial hypertension exhibited a J-shaped curve with the highest proportions in individuals with higher HR. In contrast, there was a steady increase in prevalence of obesity with increasing HR (starting from 55 bpm). No clear pattern was found for smoking, but also dyslipidemia and family history of MI. Regarding concomitant CV disease, subjects with CHF and peripheral artery disease (PAD) again showed a J-shaped curve. Individuals with CAD and MI had the greatest disease prevalence with decreased HR. For subjects with AF and stroke, no strong fluctuation was observed with varying HR.

### Age- and sex-specific distribution of heart rate in the population

We observed no resting HR age dependency for either sex in the total sample with a mean HR of 68 bpm for men and 70 bpm for women (Online Resource 4). These characteristics were also present in the subsample of individuals without intake of medications. In the subsample of individuals with healthy status, there was a difference in HR between males and females with a mean HR of 65 bpm in men and 69 bpm in women, and lower HR in men over all percentiles.

### Heart rate and all-cause mortality

**Fig. 1:**
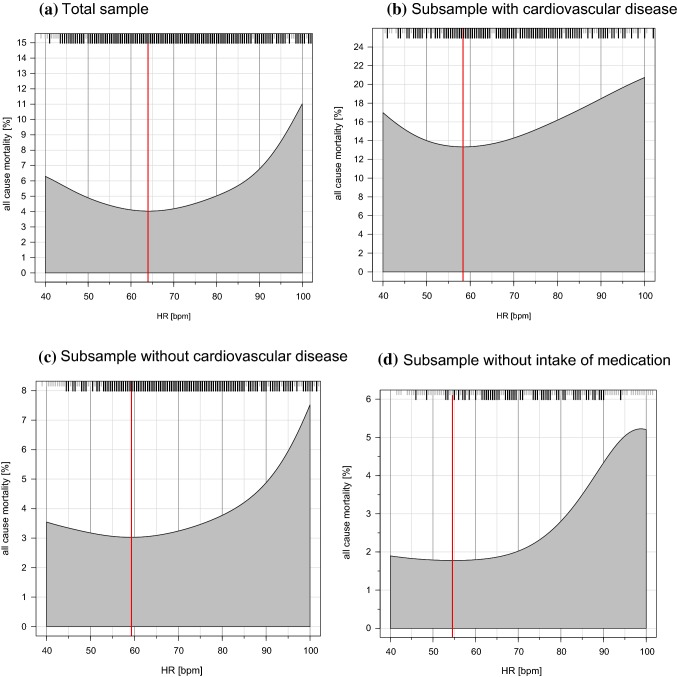
Conditional density plots presenting the relation between heart rate in beats per minute and mortality rate in % in the total and subsamples. Black stripes indicate death events and red lines indicate lowest mortality rate in relation to heart rate for **a** 64, **b** 58, **c** 59, and **d** 55. Sample sizes and number of deaths were for **a***N* = 14,925 and 714 deaths, **b***N* = 1432 and 214 deaths, **c***N* = 13,233 and 471 deaths, and **d***N* = 3517 and 82 deaths

Overall, there were 714 deaths in the study sample during a mean follow-up period of 7.67 ± 1.68 years. The conditional density plot showed a U-shaped relation between HR and all-cause mortality in the total sample (Fig. 1) with the lowest mortality rate at a HR of 64 bpm. This relation was also present in an attenuated manner in the subsamples with CV and without CV diseases with the lowest mortality rate at a HR of 58 bpm and 59 bpm, respectively. The subsample of individuals without intake of medications had an almost steady increase in mortality rates with higher HR and the lowest mortality rate at a HR of 55 bpm.

To evaluate how the prevalence of CV risk factors and concomitant diseases might influence the observations, we decided to analyze separately multivariable models below and above the nadir of HR representing the minimum of mortality (at 64 bpm) in the population sample. As indicated, there was a U-shaped relationship between HR and all-cause mortality with the lowest observed mortality rate at a nadir of 64 bpm. In preliminary analyses, 12 ATC medication classes were significantly associated with HR, so they were added to the multivariable models (Online Resource 5). In Cox regression analyses modeling for death with adjustment for age, sex, CV risk factors, concomitant diseases, and HR-associated medication, a HR below the nadir of 64 bpm was associated with a 36% increased risk of death per 10 bpm decrease, while a HR above 64 bpm was associated with a 30% increased risk of death per 10 bpm increase (Table 2).

**Table 2:** Heart rate as an independent predictor for all-cause mortality

	Adjustment for age and sex		Additional adjustment for cardiovascular risk factors and concomitant diseases		Additional adjustment for medication	
	Hazard ratio per 10 bpm (95% CI)	*P* value	Hazard Ratio per 10 bpm (95% CI)	*P* value	Hazard ratio per 10 bpm (95% CI)	*P* value
Heart rate below 64 bpm	1.54 (1.22; 1.95)	**0.00029**	1.44 (1.12; 1.85)	**0.0039**	1.36 (1.06; 1.76)	**0.016**
Heart rate above 64 bpm	1.35 (1.25; 1.46)	< **0.0001**	1.30 (1.20; 1.41)	< **0.0001**	1.30 (1.19; 1.41)	< **0.0001**

Hazard ratios and 95% confidence intervals were derived from a Cox proportional hazard regression model. Death from all causes was the dependent variable and heart rate (modeled per 10 beats per minute) the independent variable. There were 714 deaths at follow-up in the total sample

Cardiovascular risk factors comprise arterial hypertension, diabetes mellitus, dyslipidemia, family history of myocardial infarction or stroke, obesity, and smoking

Concomitant diseases were atrial fibrillation, chronic heart failure, coronary artery disease, myocardial infarction, stroke, peripheral artery disease, chronic obstructive pulmonary disease, and chronic kidney disease

Medication includes 12 medication classes from ATC taken at the time of examination, which were associated with heart rate (see Online Resource 5)

### Cardiovascular risk correlates of heart rate

**Fig. 2:**
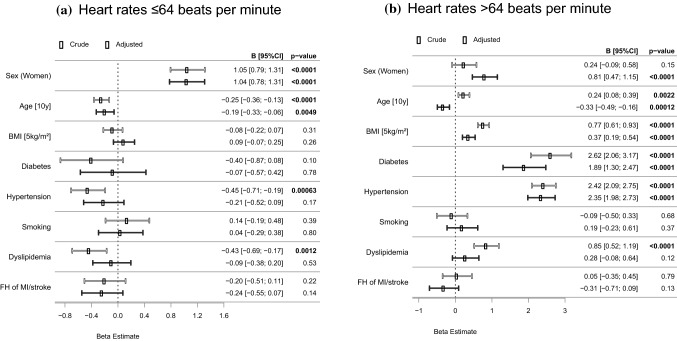
Correlates of heart rate. Beta estimates with 95% confidence intervals were derived from a linear regression model modeling for heart rates **a** ≤ 64 and **b** > 64 beats per minute as dependent variable and cardiovascular risk factors as independent variables. The crude model denotes a univariate model (white) and the adjusted model (black) denotes a multivariable model including all listed variables as covariates

To assess clinically relevant CV risk correlates of HR, we used linear regression analyses modeling for HR (Fig. 2). Focusing on HR below the nadir (≤ 64 bpm), multivariable models indicated, lower age and female sex were independently associated with HR. Analyses of individuals with HR above 64 bpm revealed lower age, female sex, higher body-mass-index, diabetes mellitus, and arterial hypertension were independently associated with HR.

### Heart rate and subclinical markers of cardiovascular disease

To further elucidate relations of subclinical markers of CV disease with HR, linear regression analyses modeling for HR with adjustment for age, sex, CV risk factors, concomitant diseases, and HR-associated medication were used (Table 3). Troponin I, CT-proAVP, NT-proBNP, and MR-proANP were independently associated with a HR of 1-SD below the nadir (< 53 bpm). CT-proAVP, MR-proADM, NT-proBNP, and MR-proANP were independently associated with a HR of 1-SD above the nadir (> 75 bpm).

**Table 3:** Heart rate and relation with subclinical markers of cardiovascular disease for (1) humoral and (2) vascular biomarkers

	Adjusted for age and sex Beta estimate (95% CI)	*P* value	Additionally adjusted for cardiovascular risk factors and concomitant diseases Beta estimate (95% CI)	*P* value	Additionally adjusted for medication Beta estimate (95% CI)	*P* value
(1) Humoral biomarkers						
*Heart rates* < *53 bpm*						
Troponin I	0.21 (0.12; 0.30)	< **0.0001**	0.19 (0.10; 0.28)	< **0.0001**	0.20 (0.11; 0.29)	< **0.0001**
Endothelin 1	− 0.013 (− 0.040; 0.015)	0.37	− 0.0088 (− 0.036; 0.018)	0.53	− 0.020 (− 0.048; 0.0071)	0.15
CT-proAVP	− 0.14 (− 0.23; − 0.059)	**0.00097**	− 0.14 (− 0.22; − 0.048)	**0.0023**	− 0.15 (− 0.23; − 0.058)	**0.0012**
MR-proADM	0.0086 (− 0.020; 0.037)	0.55	0.024 (− 0.0017; 0.050)	0.067	0.0078 (− 0.018; 0.034)	0.55
NT-proBNP	0.44 (0.30; 0.58)	< **0.0001**	0.39 (0.25; 0.53)	< **0.0001**	0.24 (0.11; 0.38)	**0.00055**
MR-proANP	0.32 (0.27; 0.37)	< **0.0001**	0.30 (0.25; 0.35)	< **0.0001**	0.22 (0.17; 0.27)	< **0.0001**
*Heart rates* > *75 bpm*						
Troponin I	− 0.023 (− 0.068; 0.022)	0.31	− 0.038 (− 0.084; 0.0075)	0.10	− 0.039 (− 0.086; 0.0073)	0.098
Endothelin 1	0.020 (0.0064; 0.034)	**0.0043**	0.0097 (− 0.0044; 0.024)	0.18	0.014 (− 0.00037; 0.028)	0.056
CT-proAVP	0.14 (0.092; 0.18)	< **0.0001**	0.10 (0.059; 0.15)	< **0.0001**	0.11 (0.069; 0.16)	< **0.0001**
MR-proADM	0.035 (0.021; 0.050)	< **0.0001**	0.0086 (− 0.0048; 0.022)	0.21	0.016 (0.0025; 0.029)	**0.020**
NT-proBNP	− 0.21 (− 0.28; − 0.14)	< **0.0001**	− 0.21 (− 0.28; − 0.14)	< **0.0001**	− 0.13 (− 0.20; − 0.056)	**0.00047**
MR-proANP	− 0.18 (− 0.20; − 0.15)	< **0.0001**	− 0.17 (− 0.20; − 0.15)	< **0.0001**	− 0.13 (− 0.16; − 0.11)	< **0.0001**
(2) Vascular biomarkers						
*Heart rates* < *53 bpm*						
Flow-mediated dilation	− 0.34 (− 0.74; 0.059)	0.095	− 0.42 (− 0.82; − 0.015)	**0.042**	− 0.41 (− 0.81; 0.00075)	0.050
Baseline brachial artery diameter	0.0086 (− 0.038; 0.055)	0.71	0.023 (− 0.022; 0.068)	0.31	0.029 (− 0.017; 0.074)	0.22
Reactive hyperemia index	0.030 (− 0.0036; 0.064)	0.080	0.018 (− 0.015; 0.051)	0.30	0.026 (− 0.0075; 0.059)	0.013
Baseline pulse amplitude	− 17.4 (− 51.6; 16.7)	0.32	− 4.79 (− 38.2; 28.6)	0.78	− 6.34 (− 40.1; 27.5)	0.71
Reflection index	7.49 (6.32; 8.67)	< **0.0001**	7.40 (6.21; 8.59)	< **0.0001**	7.45 (6.24; 8.66)	< **0.0001**
Augmentation index	15.9 (14.4; 17.4)	< **0.0001**	15.5 (14.0; 16.9)	< **0.0001**	15.2 (13.7; 16.7)	< **0.0001**
Stiffness index	− 0.67 (− 0.83; − 0.51)	< **0.0001**	− 0.65 (− 0.82; − 0.49)	< **0.0001**	− 0.58 (− 0.74; − 0.41)	< **0.0001**
*Heart rates* > *75 bpm*						
Flow-mediated dilation	− 0.012 (− 0.21; 0.18)	0.90	0.19 (− 0.0058; 0.39)	0.057	0.22 (0.017; 0.42)	**0.033**
Baseline brachial artery diameter	0.048 (− 0.018; 0.027)	0.68	− 0.037 (− 0.060; − 0.015)	**0.00097**	− 0.043 (− 0.065; − 0.020)	**0.00023**
Reactive hyperemia index	− 0.020 (− 0.036; − 0.0030)	**0.021**	− 0.00012 (− 0.017; 0.016)	0.99	− 0.0038 (− 0.021; 0.013)	0.65
Baseline pulse amplitude	− 7.27 (− 24.1; 9.58)	0.40	− 26.2 (− 42.8; − 9.63)	**0.0020**	− 26.2 (− 43.1; − 9.31)	**0.0024**
Reflection index	− 8.15 (− 8.70; − 7.59)	0.11	− 8.34 (− 8.91; − 7.77)	< **0.0001**	− 8.48 (− 9.06; − 7.90)	< **0.0001**
Augmentation index	− 11.1 (− 11.8; − 10. 3)	< **0.0001**	− 10.9 (− 11.7; − 10.2)	< **0.0001**	− 10.8 (− 11.6; − 10.1)	< **0.0001**
Stiffness index	0.23 (0.15; 0.31)	< **0.0001**	0.17 (0.090; 0.25)	< **0.0001**	0.12 (0.044; 0.20)	**0.0023**

Beta estimates and 95% confidence intervals were derived from a linear regression model modeling for heart rates below 53 (versus above 53) and above 75 (versus below 75) beats per minute (dependent variables) and with biomarkers as independent variables

Cardiovascular risk factors comprise arterial hypertension, diabetes mellitus, dyslipidemia, family history of myocardial infarction or stroke, obesity, and smoking

Concomitant diseases were atrial fibrillation, chronic heart failure, coronary artery disease, myocardial infarction, stroke, peripheral artery disease, chronic obstructive pulmonary disease, and chronic kidney disease

Medication includes 12 medication classes from ATC taken at the time of examination, which were associated with heart rate (see Online Resource 5)

Regarding vascular function markers, FMD (borderline significant *p* = 0.050), RI, AI, and SI were independently associated with a HR below 53 bpm. These parameters were also independently associated with a HR above 75 bpm. Furthermore, the structural vessel markers, baseline brachial artery diameter and baseline pulse amplitude, were associated with a HR above 75 bpm.

## Discussion

The results of the present investigation demonstrate for the first time that in a large population-based cohort, in addition to a higher HR, a lower HR is associated with increased all-cause mortality after adjustment for age, sex, CV risk factors, concomitant disease, and HR-associated medication. We found a J-shaped relationship between HR and concomitant CV disease such as CHF, CAD, PAD, and previous MI. Moreover, the relations of HR with a large panel of vascular and humoral biomarkers exhibited a differential picture when distinguishing between a lower and higher HR.

### Heart rate, cardiovascular risk factors, and concomitant cardiovascular disease

With the present study, we did not detect any dependency of age on HR in men and women, neither in the total sample, or the healthy subsample. This conflicts with previous observations, where a progressive decrease in HR over time with aging has been reported [19, 20]. As demonstrated before, women have a HR being 3–4 bpm higher compared to men [3]. This difference operates at any age, but does not increase with age. About 5% of HR was above 87 bpm in men and 88.5 bpm in women, and below 51.5 in men and 54.5 in women. These values are interestingly around 7–9 bpm lower as shown in the Framingham Study, where 5% of HR were above 95 bpm and below 60 bpm [3]. The reasons for the quite substantial reduction of almost 10 bpm in our cohort may be that study subjects of the GHS are more physical active or that more patients in the GHS have been treated with HR-lowering medication such as beta-receptor blockers having negative chronotropic effects. The observed difference in HR between men and women may be also explained, e.g., by a more on prevalence of CV risk factors [21]. Men, however, had a higher blood pressure over the entire HR range, were more likely to be smokers, obese, and diabetic. Men had higher triglyceride and lower high-density lipoprotein levels. The prevalence of subjects with previous MI, established CAD, and CHF was higher in men compared to women, while AF was more prevalent in women. In general, conditional density plots revealed that patients with CAD, previous MI, CHF, and PAD clearly had a J-shaped curve with highest prevalence in the lowest and highest HR groups, respectively.

### Heart rate and relations with hemodynamic, vascular, and humoral biomarkers

Resting HR has been consistently demonstrated to be associated with arterial blood pressure in epidemiologic [19, 20], but also pathophysiological studies [22, 23]. In the general population, this relationship between HR and blood pressure was confirmed over the whole range of blood pressure values and has been observed at any age [22]. In the present study, we could observe an increase in systolic blood pressure 1-SD below and above the nadir of HR, but also a steady increase in diastolic and mean arterial pressure accompanied by a steady increase in arterial stiffness (as indicated by SI) over all HR groups. These results are in line with previous observations demonstrating that, e.g., increasing HR in patients with implanted pacemaker will, with a higher HR, increase blood pressure and simultaneously increase arterial stiffness [24, 25].

Regarding humoral biomarkers, NT-proBNP, and MR-proANP showed a clear decrease over the HR groups, while other markers did not show a clear pattern. When comparing groups of HR (i.e., <53 bpm and > 75 bpm) in linear regression analysis, CT-proAVP, NT-proBNP and MR-proANP remained independently associated in both HR groups, but in an inverse relation. With respect to vascular biomarkers, increased HR from below 55 bpm up to above 75 bpm was accompanied by an increase in FMD, RHI, and SI, while RI and AI decreased concordantly. However, linear regression models revealed reduced FMD and SI as well as increased RI and AI to be independently associated with a HR below 53 bpm, while these markers were also independently related to a HR above 75 bpm, but again in an inverse relation.

The pattern of results found for humoral and vascular biomarkers clearly exhibited a differential picture in subjects with a HR below 53 bpm and above 75 bpm, suggesting that different pathophysiological mechanisms may be responsible for the relation of HR with mortality as indicated by the inverse relation of markers. The present analyses cannot fully clear at this point since further mechanisms may be involved in the interplay between HR and these markers. Clearly, a higher HR is directly associated with autonomic imbalance and thus a hyperactive sympathetic and a hypoactive parasympathetic system. Nevertheless, it is still difficult to specify HR-mediated effects of autonomic imbalance and to distinguish from related effects, in particular for lower HR [26]. However, increased troponin I, NT-proBNP, and MR-proANP levels as well as reduced FMD and increased RI and AI were independently associated with a HR below 53, which may demonstrate relevant pathways for understanding the relationship between lower HR and mortality, whereas the association with higher HR may be more affected by an increased prevalence of CV risk factors.

### Heart rate and all-cause mortality

With the present study, we showed an increase in mortality rates with higher HR, as it has been shown before [27], but also an increase in mortality in patients with lower HR. The nadir of HR, where the lowest mortality was seen was 64 bpm. The observation of a J-shaped mortality curve is contrasting previous reports from the Framingham Study where mortality increases progressively with resting HR [3]. Likewise, in the Goteborg Primary Prevention Trial [28] and the NHEFS Cohort [29], the rate of death from all causes and CV disease increased as a function of increasing HR or when pulse rate increased beyond 84 bpm. Since beta-receptor blockade was in the late 80s not established yet for the treatment of patients with CHF, it is tempting to speculate that the J-shaped curve seen in the present study rather reflects a drug effect. However, we observed a J-shaped relation between HR and mortality in the subsample without CV disease and further adjusted for concomitant disease as well as HR-associated medication (including beta-receptor blockers) in Cox regression analysis for mortality.

### Optimal heart rate in patients with coronary artery disease and chronic heart failure

Although there is no doubt that patients with CAD and CHF will have symptomatic and/or prognostic benefit from a reduction in HR being higher than 70 bpm [2, 4, 5, 11], the question remains whether the clinical benefit of a HR reduction may be turned into the opposite when the HR reduction, e.g., is too pronounced. The results of the present study suggest that a HR below 64 bpm may increase rather than further decrease mortality. These findings may explain at least in part why the recent SIGNI_f_Y Trial [11] failed to demonstrate beneficial effects on patients with stable CAD having activity limiting angina and a HR higher than 70 bpm. By adding ivabradine to baseline beta-receptor blocker therapy, the patients had a quite substantial HR reduction of about 8 bpm, but also high numbers of side effects such as symptomatic bradycardia and in the subgroup of patients with limiting angina even more CV event rates [11]. Hereby, the J-shaped relationship between HR and outcomes as observed in the present study would be one possible explanation.

### Strengths and limitations

To our knowledge, this is the first study that evaluated the association between higher but also lower HR and a large panel of markers suggestive of CV risk as well as mortality in a population-based setting. Strengths of the present study include the detailed characterization of age- and sex-specific distribution of HR in the general population. Further, the large sample size of the GHS cohort across a broad age spectrum as well as the comprehensive and standardized assessment of multiple biomarkers, CV risk factors, concomitant disease, and further included variables are notable. Some limitations, however, need to be considered. A major limitation of the present analysis is the lack of information on the cause of death. We cannot differentiate between CV and non-CV causes. However, since almost half of mortality cases are due to CV causes [30], we still have good power to detect clinically relevant associations. Although the mortality data were derived from a prospective design, results must be considered with caution in terms of causation given the observational and non-randomized nature of the study. We cannot fully exclude, for example, that altered HR may be simply an epi-phenomenon, resulting from a hidden and unmeasured confounding etiological cause, leading to increased mortality. Also, we cannot differentiate between specific drug effects as we solely considered HR-associated medication by class, which may have affected our results. For example, it is known that HR reduction induced by beta-receptor blockers is complex as a result of autonomic modulations, while HR reduction is the only CV effect of ivabradine [31, 32]. Moreover, the mortality analyses were based on a single baseline measurement of HR and thus may be more sensitive to fluctuations.

## Summary and conclusions

The results of the present study show a higher prevalence in most of CV risk factors with a higher HR. In patients with concomitant CV disease such as CHF, CAD, PAD, and previous MI, there is a clear J-shaped relationship with HR. In this population-based cohort, the all-cause mortality curve revealed a nadir of HR at 64 bpm with an increase in mortality above but also below the nadir. The study may also indicate that patients with CHF may not benefit from a too strong HR reduction. Further studies are warranted to get more insight in the pathophysiological mechanisms underlying the relationship between a low HR and mortality.

## Electronic supplementary material

Below is the link to the electronic supplementary material.

Supplementary material 1 (DOCX 1062 KB)
