# Supplementary material for: Heart rate, mortality, and the relation with clinical and subclinical cardiovascular diseases: results from the Gutenberg Health Study
Source: Clin Res Cardiol. 2019 Apr 5;108(12):1313–23. doi: 10.1007/s00392-019-01466-2 (PMC6868108; doi:10.1007/s00392-019-01466-2)
Supplement: Supplementary file 1 — Supplementary material 1 (DOCX 1062 KB) [file 392_2019_1466_MOESM1_ESM.docx]

**Heart rate, mortality, and the relation with clinical and subclinical cardiovascular diseases: results from the Gutenberg Health Study**

**Online supplementary material**

Thomas Münzel, MD^1,2,3^; Omar Hahad, PhD^1^; Tommaso Gori, MD, PhD^1,3^; Sebastian Hollmann, MD^1^; Natalie Arnold, MD^1,4^; Jürgen H. Prochaska, MD^2,3,4^; Andreas Schulz, PhD^4,^; Manfred Beutel, MD^5^; Norbert Pfeiffer, MD^6^; Irene Schmidtmann, PhD^7^; Karl J. Lackner, MD^3,8^; John F. Keaney, Jr., MD^9^; Philipp S. Wild, MD, MSc ^2,3,4^

^1^ Center for Cardiology – Cardiology I, University Medical Center of the Johannes Gutenberg-University Mainz, Mainz, Germany

^2^ Center for Thrombosis and Hemostasis, University Medical Center of the Johannes Gutenberg-University Mainz, Mainz, Germany

^3^ German Center for Cardiovascular Research (DZHK), partner site RhineMain, Mainz, Germany

^4^ Preventive Cardiology and Preventive Medicine, Center for Cardiology, University Medical Center of the Johannes Gutenberg-University Mainz, Mainz, Germany

^5^ Department of Psychosomatic Medicine and Psychotherapy, University Medical Center of the Johannes Gutenberg-University Mainz, Mainz, Germany

^6^ Department of Ophthalmology, University Medical Center of the Johannes Gutenberg-University Mainz, Mainz, Germany

^7^ Institute of Medical Biostatistics, Epidemiology & Informatics, University Medical Center of the Johannes Gutenberg-University Mainz, Mainz, Germany

^8^ Institute of Clinical Chemistry and Laboratory Medicine, University Medical Center of the Johannes Gutenberg-University Mainz, Mainz, Germany

^9^ Division of Cardiovascular Medicine, University of Massachusetts Medical School, Worcester, Massachusetts, USA

**Address for correspondence:**

Professor Thomas Münzel, MD

University Medical Center of the Johannes Gutenberg-University Mainz

Center for Cardiology – Cardiology I

Langenbeckstraße 1, 55131 Mainz, Germany

Phone: +49 (0) 6131 17-7250

Fax: +49 (0) 6131 6615

Email: [tmuenzel@uni-mainz.de](mailto:tmuenzel@uni-mainz.de)

This supplementary material has been provided by the authors to give readers additional information.

**
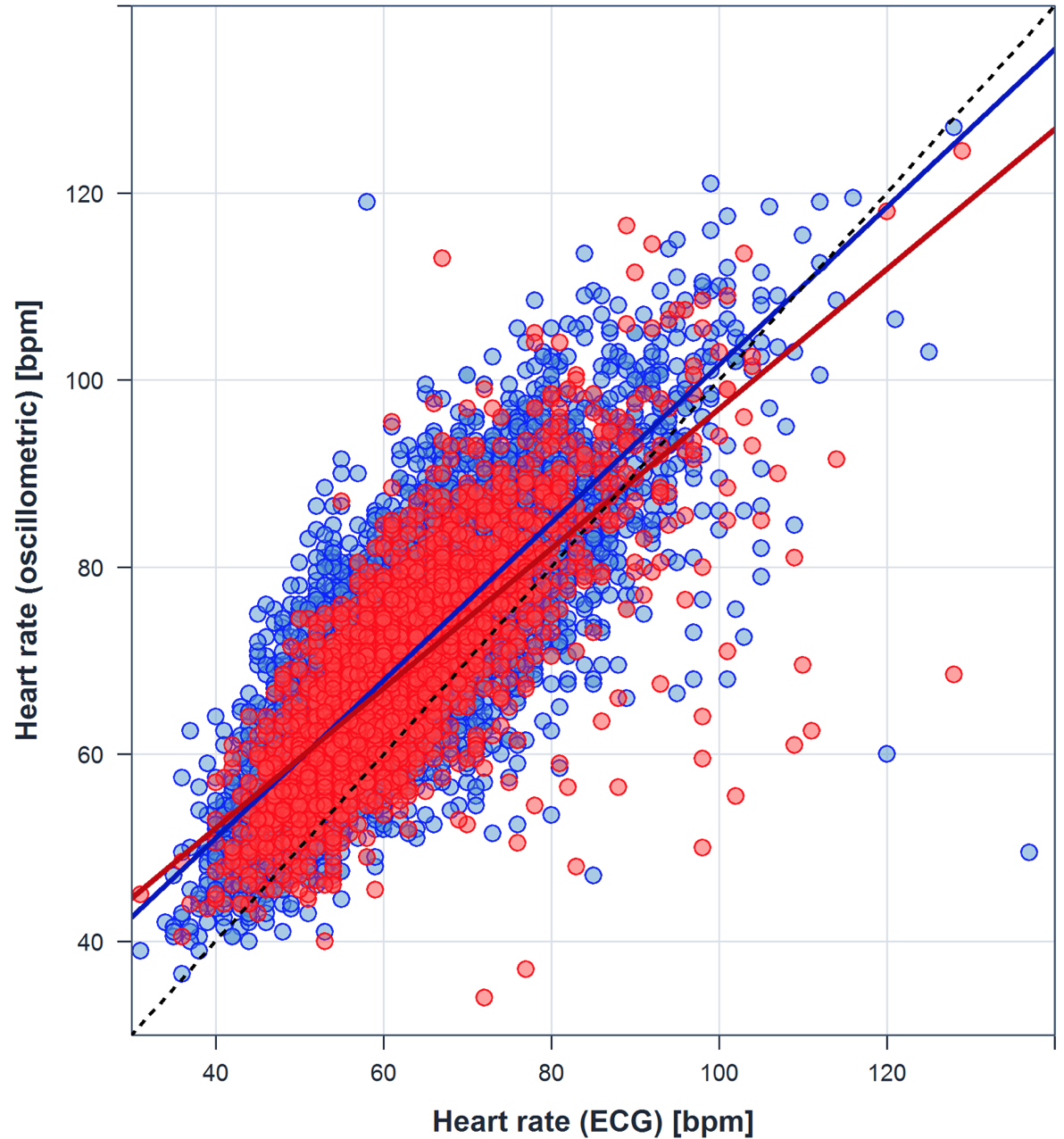
**

**Online Resource 1** Scatter-plot presenting the product-moment correlation between oscillometric- and ECG-based measurement of heart rate in beats per minute in subjects with atrial fibrillation (marked in red; *r*=0.76; 95% confidence interval 0.74-0.77) and in the total sample (cases without atrial fibrillation are marked in blue; *r*=0.79; 95% confidence interval 0.79-0.80)

(a) Diabetes mellitus (b) Arterial hypertension

(c) Obesity (d) Smoking

(e) Dyslipidemia (f) Family history of myocardial infarction/stroke

**Online Resource 2** Heart rate and prevalence of cardiovascular risk factors. Conditional density plots presenting the prevalence in % of (a) diabetes mellitus, (b) arterial hypertension, (c) obesity, (d) smoking, (e) dyslipidemia, and (f) family history of myocardial infarction or stroke according to heart rate in beats per minute

(a) Chronic heart failure (b) Peripheral artery disease

(c) Coronary artery disease (d) Myocardial infarction

(e) Atrial fibrillation (f) Stroke

**Online Resource 3** Heart rate and prevalence of cardiovascular disease. Conditional density plots presenting the prevalence in % of (a) chronic heart failure, (b) peripheral artery disease, (c) coronary artery disease, (d) myocardial infarction, (e) atrial fibrillation, and (f) previous stroke according to heart rate in beats per minute

**Online Resource 4** Age- and sex-specific distribution of heart rate in the population

(1) Total sample

|  | *Age intervals* | | | |  |
| --- | --- | --- | --- | --- | --- |
|  | 35-44 | 45-54 | 55-64 | 65-74 | Total |
|  | *Men* | | | |  |
| *Mean (SD)* | 68.2 (10.6) | 68.5 (10.6) | 68.4 (11.3) | 66.8 (11.7) | 68.0 (11.1) |
| *Median (Q1/Q3)* | 67.5 (61.0/75.0) | 67.8 (61.5/75.0) | 67.5 (60.5/76.0) | 65.5 (59.0/73.5) | 67.0 (60.5/75.0) |
| *2.5% (P)* | 49.5 | 49.5 | 49.0 | 47.5 | 48.5 |
| *5%* | 52.5 | 53.0 | 52.0 | 50.0 | 51.5 |
| *95%* | 86.5 | 87.0 | 88.0 | 87.5 | 87.0 |
| *97.5%* | 91.5 | 92.5 | 93.0 | 93.5 | 93.0 |
|  | *Women* | | | |  |
| *Mean (SD)* | 70.7 (10.2) | 69.7 (10.3) | 69.4 (10.5) | 70.3 (10.8) | 70.0 (10.5) |
| *Median (Q1/Q3)* | 70.0 (64.0/76.5) | 69.0 (63.0/76.0) | 69.0 (62.0/76.0) | 69.5 (62.5/77.0) | 69.5 (63.0/76.0) |
| *2.5% (P)* | 52.5 | 51.2 | 50.8 | 52.5 | 52.0 |
| *5%* | 55.5 | 54.0 | 54.0 | 54.5 | 54.5 |
| *95%* | 88.5 | 88.0 | 87.5 | 89.2 | 88.5 |
| *97.5%* | 93.0 | 93.0 | 92.5 | 94.5 | 93.4 |

(2) Individuals without intake of medication

|  | *Age Intervals* | | | |  |
| --- | --- | --- | --- | --- | --- |
|  | 35-44 | 45-54 | 55-64 | 65-74 | Total |
|  | *Men* | | | |  |
| *Mean (SD)* | 68.7 (9.97) | 68.3 (9.95) | 69.1 (10.2) | 69.3 (10.8) | 68.7 (10.1) |
| *Median (Q1/Q3)* | 68.0 (62.0/74.5) | 67.5 (62.0/74.5) | 69.0 (61.5/76.5) | 68.5 (62.3/75.2) | 68.0 (62.0/75.0) |
| *2.5% (P)* | 50.1 | 49.9 | 50.0 | 49.5 | 50.0 |
| *5%* | 55.0 | 53.0 | 52.8 | 53.0 | 53.5 |
| *95%* | 86.5 | 85.0 | 85.0 | 88.3 | 86.0 |
| *97.5%* | 89.0 | 89.5 | 89.0 | 92.3 | 89.5 |
|  | *Women* | | | |  |
| *Mean (SD)* | 68.5 (10.7) | 68.7 (10.8) | 68.4 (10.0) | 70.0 (11.6) | 68.7 (10.7) |
| *Median (Q1/Q3)* | 67.5 (61.5/75.0) | 67.5 (61.5/75.0) | 68.0 (61.0/75.0) | 69.0 (63.5/76.8) | 67.5 (61.5/75.0) |
| *2.5% (P)* | 51.0 | 49.5 | 51.0 | 50.5 | 50.5 |
| *5%* | 53.0 | 52.5 | 53.5 | 51.7 | 53.0 |
| *95%* | 86.5 | 87.8 | 85.5 | 88.3 | 87.0 |
| *97.5%* | 93.0 | 93.0 | 90.3 | 100 | 92.9 |

(3) Individuals with healthy status

|  | *Age intervals* | | | |  |
| --- | --- | --- | --- | --- | --- |
|  | 35-44 | 45-54 | 55-64 | 65-74 | Total |
|  | *Men* | | | |  |
| *Mean (SD)* | 65.8 (9.35) | 64.6 (9.04) | 64.8 (8.69) | 64.1 (9.06) | 65.0 (9.11) |
| *Median (Q1/Q3)* | 64.5 (59.0/72.0) | 64.0 (59.0/70.0) | 64.0 (58.0/71.0) | 64.0 (58.9/68.8) | 64.5 (59.0/70.5) |
| *2.5% (P)* | 49.5 | 46.7 | 49.0 | 44.4 | 48.3 |
| *5%* | 52.0 | 50.8 | 50.9 | 49.4 | 51.0 |
| *95%* | 81.9 | 80.8 | 80.0 | 82.0 | 81.0 |
| *97.5%* | 84.5 | 82.5 | 83.3 | 84.1 | 84.0 |
|  | *Women* | | | |  |
| *Mean (SD)* | 69.6 (9.66) | 68.6 (8.82) | 67.8 (9.29) | 69.9 (8.16) | 69.0 (9.23) |
| *Median (Q1/Q3)* | 68.5 (63.0/75.5) | 68.0 (63.0/74.0) | 68.0 (61.0/73.5) | 68.5 (64.6/74.5) | 68.5 (63.0/74.5) |
| *2.5% (P)* | 52.5 | 52.6 | 50.5 | 54.6 | 52.5 |
| *5%* | 55.0 | 54.5 | 53.7 | 58.5 | 54.9 |
| *95%* | 87.0 | 83.0 | 82.8 | 84.0 | 84.5 |
| *97.5%* | 90.9 | 86.5 | 86.0 | 86.8 | 89.0 |

Distribution in the total sample ((1) *N*=15,010), subsample of individuals without intake of medication ((2) *N*=3,517), and subsample of individuals with healthy status ((3) *N*=2,224). The healthy subsample was defined as individuals without cardiovascular risk factors (i.e. without diabetes mellitus, arterial hypertension, smoking, obesity, dyslipidemia, and family history of myocardial infarction/stroke) and without concomitant diseases (i.e. chronic heart failure, coronary artery disease, myocardial infarction, stroke, atrial fibrillation, peripheral artery disease, chronic kidney disease, chronic liver disease, and chronic obstructive pulmonary disease)

*P* percentile, *SD* standard deviation, *Q* quartile

| **Online Resource 5** Heart rate-associated medication classes of the Anatomical Therapeutic Chemical Classification | | |
| --- | --- | --- |
| 3-digit categories | Adjustment for age and sex  Beta estimate per SD (95% CI) | *P*-value |
| A10 (Drugs used in diabetes) | 0.80 (0.62; 0.98) | **<0.0001** |
| C10 (Lipid modifying agents) | -0.38 (-0.57; -0.20) | **<0.0001** |
| C07 (Beta blocking agents) | -1.99 (-2.17; -1.81) | **<0.0001** |
| B01 (Antithrombotic agents) | -0.55 (-0.74; -0.36) | **<0.0001** |
| R03 (Drugs for obstructive airway diseases) | 0.67 (0.49; 0.84) | **<0.0001** |
| N06 (Psychoanaleptics) | 0.35 (0.17; 0.52) | **<0.0001** |
| H02 (Corticosteroids for systemic use) | 0.32 (0.15; 0.49) | **0.00032** |
| A02 (Drugs for acid related disorders) | 0.29 (0.11; 0.46) | **0.0015** |
| N05 (Psycholeptics) | 0.28 (0.11; 0.46) | **0.0016** |
| R05 (Cough and cold preparations) | 0.28 (0.10; 0.45) | **0.0017** |
| M04 (Antigout preparations) | 0.27 (0.098; 0.45) | **0.0024** |
| R01 (Nasal preparations) | 0.24 (0.065; 0.41) | **0.0072** |

Beta estimates and 95% confidence intervals were derived from a linear regression model for heart rate. Medication classes include all ATC categories taken at the time of examination with more than 25 subjects. Only medication classes with a significant association (*p*-value <0.01) with heart rate are displayed
